# Microscopy observations reveal a new glandular morphology in four *Pinguicula* L. species

**DOI:** 10.1186/s13104-024-07021-1

**Published:** 2024-12-19

**Authors:** Sara Natale, Marco Canella, Silvia Moschin, Barbara Baldan, Alessandro Alboresi, Nicoletta La Rocca, Francesco Dal Grande

**Affiliations:** 1 Department of Biology, University of Padova, 35123 Via Ugo Bassi 58/B, Padova, Italy; 2 National Biodiversity Future Center, Palermo, Italy; 3 Botanical Garden of Padova, 35123 Via Orto Botanico 15, Padova, Italy

**Keywords:** Carnivorous plants, Lentibulariaceae, Scanning electron microscopy, Micromorphology, Glands, Trichomes, Pinguicula, Butterwort

## Abstract

The adaxial leaf surface of butterworts (*Pinguicula* L.) presents specialized structures for carnivory, such as trichomes and sessile glands. The micromorphology of abaxial leaf surfaces has rarely been investigated; therefore, this study aimed to compare the micromorphology of adaxial and abaxial surfaces through electron scanning microscopy (SEM) and light microscopy (LM). We selected four species occurring in the Eastern Alps. All the species showed the same pattern: trichomes and sessile glands occurring only on the adaxial surface; stomata occurring on both surfaces with significant differences in density and size; and four-cells glands occurring only on the abaxial surface. The four-cells gland is a micromorphological structure that has never been described before, and its eco-physiological function needs to be investigated.

**Supplementary Information:**

The online version contains supplementary material available at 10.1186/s13104-024-07021-1.

## Introduction

*Pinguicula* L. (butterworts) is a genus belonging to the Lentibulariaceae family that includes several genera of carnivorous plants, including *Utricularia* L. (bladderworts) and *Genlisea* A.St.-Hil. (corkscrew plants). The genus *Pinguicula* comprises approximately 100 species [1] and is primarily distributed in temperate and tropical regions of the Northern Hemisphere, including Europe, North and South America, and parts of Asia and Africa [2]. Carnivorous traits have independently evolved multiple times within the Lentibulariaceae family, resulting in diverse trapping mechanisms [3]. In *Pinguicula*, the leaves are characterized by stalked glandular trichomes (hereafter referred to as “trichomes”) and sessile glands. Stalked structures protrude from the adaxial surface of the leaf and are fundamental for producing mucilage to attract and capture insects [4]. The sessile glands produce a clear and viscous mucilage that coats the surface of the leaf and digests the prey. Nonglandular trichomes have also been found in *Pinguicula* and are likely involved in the pollination syndrome of some species of Central-South America [5]. The trapping mechanism of *Pinguicula* allows the absorption of essential nutrients such as nitrogen and phosphorus, which are typically lacking in their native habitats (see 6 for an extensive morphological description of secretory glands in *Pinguicula*). *Pinguicula* typically occurs in moist and nutrient-poor environments such as bogs and rocky areas. In this study, we considered four species of *Pinguicula* occurring in the montane and alpine environment of the Eastern Alps [7]. We applied light microscopy (LM) and scanning electron microscopy (SEM) to compare the leaf micromorphology of the adaxial and abaxial surfaces. The adaxial surface of different species of *Pinguicula* has been the subject of many morphological, micromorphological, and cytochemical studies (see, e.g., [7–9]); however, to the best of our knowledge, the abaxial surface has barely been characterized. By filling this gap, we aim to provide a morphological basis for better understanding the ecological and physiological processes that occur between the abaxial surface and the growing environment.

## Materials and methods

The study species were *Pinguicula alpina* L., *P. poldinii* J. Steiger & Casper, *P. leptoceras* Rchb. and *P. vulgaris* L. Each species was sampled in its natural habitat during the 2023 growing season. The sampling locations and growing environments are reported in Table 1.

**Table 1:** List of sampled *Pinguicula* species along with their sampling locations, infra-generic clade, distribution, and growing environment

Species	Latitude	Longitude	Subgenus	Distribution	Growing environment
*P. alpina*	46.18571°	12 0.04401°	Temnoceras	Northern Hemisphere	Damp limestone-rich cliffs and crags
*P. poldinii*	46.07859°	11 0.87678°	Orcheosanthus	Endemic to North-Eastern Italy	Damp limestone-rich cliffs and crags
*P. leptoceras*	46.09491°	11 0.84132°	Temnoceras	Alpine Arc	Mountain damp meadows
*P. vulgaris*	46.47659°	12 0.61054°	Temnoceras	Northern Hemisphere	Mountain damp meadows

Leaves from five different individuals per species were fixed in a mixture of 3.5% glutaraldehyde with 2.5% formaldehyde in 0.05 M cacodylate buffer, pH 7.2 (Sigma Aldrich^®^), and transferred to the laboratory in a cool bag. Once in the laboratory, the samples were incubated overnight at 4 °C [4]. The samples were then dehydrated in solutions with increasing concentrations of ethanol (20%, 50%, 80%, 90%, 100%) and subjected to critical point drying using CO_2_. After gold coating, the adaxial and abaxial surfaces of the leaves were examined with a JSM Jeol 6490 scanning electron microscope (SEM) at an accelerating voltage of 20 kV. For each species, we verified the presence or absence of trichomes, glands and stomata. We measured the following traits on both the adaxial and abaxial surfaces (10 replicates) using ImageJ software (http://imagej.nih.gov/ij/*)*: average trichome diameter (head), gland diameter, and stomatal length (major axis). We further calculated the density of trichomes, glands, and stomata in an area of 0.3 mm^2^ for three replicates. Statistical analysis of the morphometric data was performed in R [10]. After checking the ANOVA assumptions, we conducted one-way ANOVA (‘aov’ function in ‘stats’ R package) followed by a post hoc comparison using Tukey’s Honestly Significant Difference test (Tukey HSD, ‘Tukey HSD’ function in ‘stats’ R package). A set of leaves, collected and fixed as described above, were embedded in Technovit 7100^®^ (Heraeus Kulzer, Germany). Sections were cut with a microtome (Leica RM2125 RTS) to obtain transverse sections. (6–7 µm thickness) and then stained with 0.1% toluidine blue. The sectioned slides were visualized using a Leica DM500 microscope equipped with a Leica ICC50 W camera for imaging (Leica Application Suite software).

## Results and discussion

**Fig. 1:**
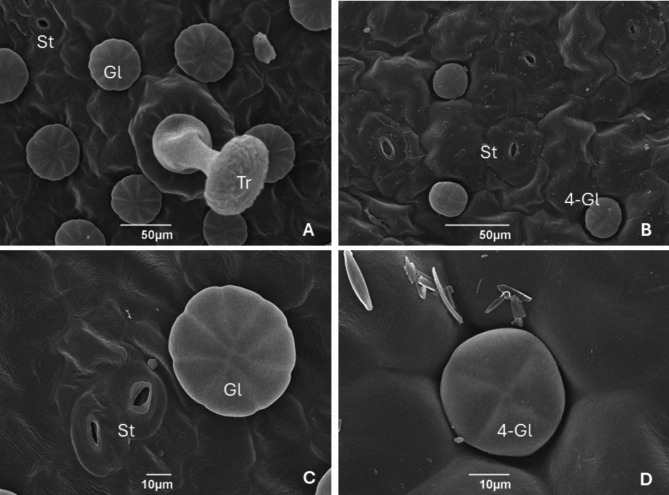
Scanning electron microscopy (SEM) images of *Pinguicula* leaves. (**A**) Adaxial surface of *P. alpina* showing a trichome, a stoma, and secretory glands; (**B**) Abaxial surface of *P. alpina* with stomata and four-cells glands; (**C**) Close-up of a secretory gland on the adaxial surface in *P. leptoceras*; (**D**) Detailed view of the four-cells gland of the abaxial surface in *P. vulgaris*. Abbreviations: St, stoma Gl, gland; Tr, Trichome; 4-Gl, four-cells gland

We observed a consistent pattern across the entire set of species studied. On the adaxial surface, we detected glandular trichomes, secretory glands, and stomata (Fig. 1A), while on the abaxial surface, we detected stomata and four-cells glands (Fig. 1B). SEM images for all the studied species are provided in the additional materials.

The morphometric characteristics of the trichomes, stomata and glands are summarized in Table 2.

**Table 2:** Mean values ± standard deviations of morphometric traits measured in the leaves of four species of *Pinguicula* L. diameters are expressed in µm, while densities are expressed as the number of structures per mm^2^. Different letters indicate significant differences between samples (*p* < 0.05, Tukey test)

Adaxial leaf surface						
Species	Gland diameter	Trichome head diameter	Stomata length	Trichome density	Gland density	Stomata density
*P. alpina*	54.76 ± 3.14 a	77.46 ± 3.26 a	39.62 ± 3.56	5.56 ± 0.74 a	79.64 ± 1.33 a	28.30 ± 5.74 ab
*P. poldini*	49.90 ± 2.98 b	64.05 ± 5.39 b	36.40 ± 2.94	7.50 ± 1.67 a	133.33 ± 13.88 b	25.00 ± 4.30 a
*P. vulgaris*	56.77 ± 4.01 a	88.64 ± 4.40 c	38.76 ± 4.07	14.17 ± 4.19 b	112.5 ± 7.35 c	39.71 ± 3.80 b
*P. leptoceras*	46.98 ± 2.20 b	75.66 ± 3.73 a	35.22 ± 5.55	16.94 ± 1.61 b	86.29 ± 9.9 9 a	25.81 ± 8.33 a
**Abaxial leaf surface**						
**Species**	**Four-cells gland diameter**	**Stomata length**	**Four-cells gland density**	**Stomata density**		
*P. alpina*	33.03 ± 2.89 a	38.41 ± 3.00	40.63 ± 6.25 a	17.6 4 ± 4.56 a		
*P. poldini*	29.42 ± 2.99 b	40.71 ± 8.45	24.52 ± 3.67 b	25.74 ± 3.70 a		
*P. vulgaris*	34.19 ± 1.99 a	40.13 ± 3.28	62.10 ± 4.06 c	45.97 ± 5.51 b		
*P. leptoceras*	33.29 ± 2.26 a	37.78 ± 3.56	35.48 ± 2.63 a	48.39 ± 4.56 b		

Stomata were found on both surfaces of the leaf. All the stomata were composed of 3 cells unequal in size surrounding the guard cells. This morphology belongs to the anisocytic type according to the Metcalfe and Chalak classification of the stoma complex [11]. In all the species and on both leaf surfaces, we sometimes found contiguous stomata. This clustering has already been shown, and it occurs when growing meristemoids differ between two guard cells ([12], Fig. 1C). The stomatal density on the adaxial surface varied from 25.00 ± 4.30 stomata/mm^2^ for *P. poldinii* to 39.71 ± 3.80 stomata/mm^2^ for *P. vulgaris*. The abaxial surface stomatal density ranged from 17.64 ± 4.56 stomata/mm^2^ in *P. alpina* to 48.39 ± 4.56 stomata/mm^2^ in *P. leptoceras*. Variations in stomatal density could be linked to environmental features of the growing habitat, such as temperature, air humidity, soil moisture, and light intensity [13, 14]. There was no significant difference in major axis length among stomata types (χ²=45.65, df = 3, *P* = 0.06).

The general morphology of trichomes did not change across all the species examined. All trichomes observed were glandular, with a compressed globular head. The length of the stalk was approximately 50 μm. *P. poldinii* exhibited smaller glandular heads (average diameter 64.05 ± 5.39 μm) and a lower density of trichomes (7.50 ± 1.67 trichomes/mm^2^). Compared with those of the other species, *P. vulgaris* (88.64 ± 4.40 μm) had significantly larger glandular heads in diameter, while *P. vulgaris* and *P. leptoceras* had greater trichome density than the other two species (χ²=114.8, df = 3, *P* < 0.05). It is known that trichomes can vary in size and morphology among species [9], and the trade-off between trichome size and prey has already been documented [15]. The differences in trichome dimensions among the studied species could suggest diversity in the size of prey, which in these areas are mainly Diptera.

Sessile glands are round shaped, and their number of cells can vary greatly (from 8 to 25 in the studied species). The diameter varies from 46.98 ± 2.20 μm in *P. leptoceras* to 56.77 ± 4.01 μm in *P. vulgaris.* Their density was significantly lower in *P. alpina* and *P. leptoceras* (80.64 ± 1.33 μm and 86.29 ± 9.99 μm, respectively) (χ²=219.8, df = 3, *P* < 0.05).

**Fig. 2:**
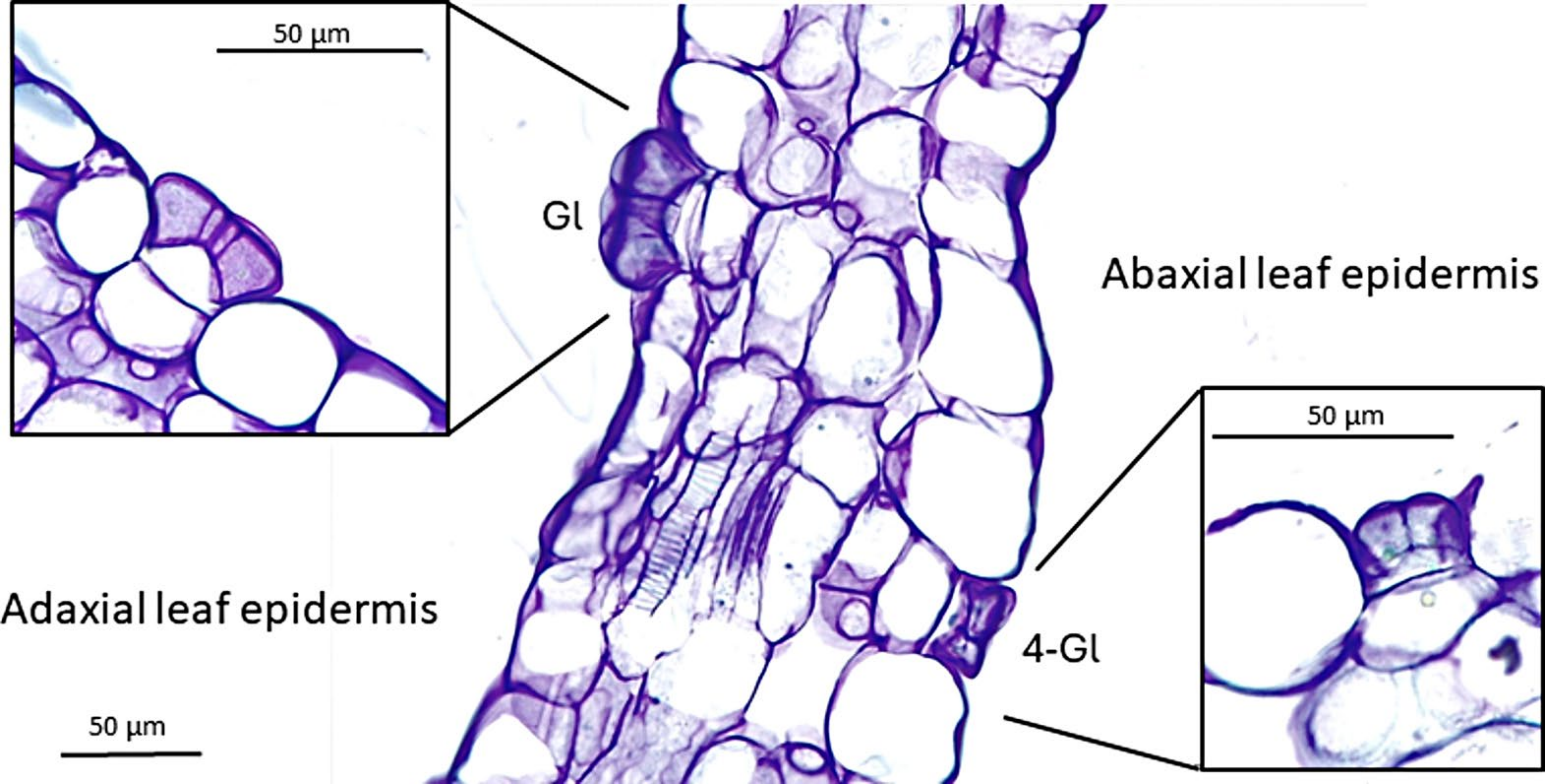
Leaf transverse sections of *P. alpina* showing the secretory gland of the adaxial surface and the four-cells gland on the abaxial surface are clearly visible. Abbreviations: Gl, gland; 4-Gl, four-cells gland

A major finding of this work concerns the glandular structures of the abaxial surface. Unlike the glands on the adaxial surface (Fig. 1C), these glands are always composed of four cells (Fig. 1D), ranging from 29.42 ± 2.99 μm in diameter for *P. poldinii* to 34.19 ± 1.99 μm for *P. vulgaris*. The general shape ranges from round to elliptical. The transverse sections employed in LM imaging allowed for clear visualization of the position of the abaxial surface glands, which are located deeper into the leaf surface than the adaxial surface secretory glands (Fig. 2).

We considered the possibility that four-cells glands might represent a “common” developmental stage of secretory glands rather than a novel morphology. Its four-cells morphology is similar to that of secretory glands in the early developmental stage [5]. However, no four-cells glands were found on the adaxial surface, and all glands on the abaxial surface were four-cells shaped. This pattern was consistent across the species studied. We argue that this previously undescribed gland morphology is exclusive to the abaxial surface of *Pinguicula*. The function of the four-cells glands needs further investigation. Lentibulariaceae species have evolved different strategies to absorb additional nutrients, such as “vegetarianism”, through the synthesis of α-amylase in *Pinguicula* [16] and *Utricularia* [17] species. *Pinguicula* has a rosette life form, with many basal leaves in contact with the soil. Thus, we can hypothesize that the four-cells glands produce mucilage that interacts with the soil biota or other potential nitrogen and phosphorus sources. Mucilages might also play a defensive role, as demonstrated in *P. moranensis* Humboldt, Bonpland & Kunth [18]. In this context, the four-cells gland could represent a defense structure against predation. The density of four-cells glands varies greatly among species, from 25.52 ± 3.67 glands/mm^2^ in *P. poldinii* to 62.10 ± 4.06 in *P. vulgaris*.

## Conclusions

Glandular trichomes, secretory glands, and stomata are present on the adaxial surface of leaves, while only stomata and a newly described glandular structure, the four-cells gland, have been found on the abaxial side. This four-cells gland occurs on the abaxial leaf surface of all four species of *Pinguicula* examined and, to the best of our knowledge, has never been described before. As a next step, the prevalence of this morphology within the genus needs to be clarified. Further studies are required to understand the function of this glandular structure, both through additional microscopy techniques (e.g., transmission electron microscopy) and metabolomic analyses. Future studies should focus also on the possible link between morphometric characteristics and environmental factors to better elucidate whether these differences are species-specific or environment related.

### Limitations

The current study is based on four species of *Pinguicula*. Although these species well represent the diversity of the genus in the Eastern Alps, additional studies are required to determine whether four-cells glands are also present in other congeneric species, e.g., tropical ones. Moreover, the eco-physiological role of these structures remains to be investigated.

## Electronic supplementary material

Below is the link to the electronic supplementary material.

Supplementary Material 1

## Data Availability

No datasets were generated or analysed during the current study.

## Abbreviations

SEM: Scanning electron microscope
LM: Light microscopy
