# Supplementary material for: Microscopy observations reveal a new glandular morphology in four Pinguicula L. species
Source: BMC Res Notes. 2024 Dec 19;17:367. doi: 10.1186/s13104-024-07021-1 (PMC11660648; doi:10.1186/s13104-024-07021-1)

**Supplementary materials**

Scanning electron microscope (SEM) images of *Pinguicula* leaves. Each plate reports from the top left, clockwise: adaxial leaf surface; abaxial leaf surface; close-up of a secretory gland on the adaxial surface; close-up of the four-cell gland on the abaxial surface.
Abbreviations: St, stomata; Gl, gland; Tr, trichome; 4-Gl, four-cell gland.

Plate 1 *Pinguicula alpina* L.


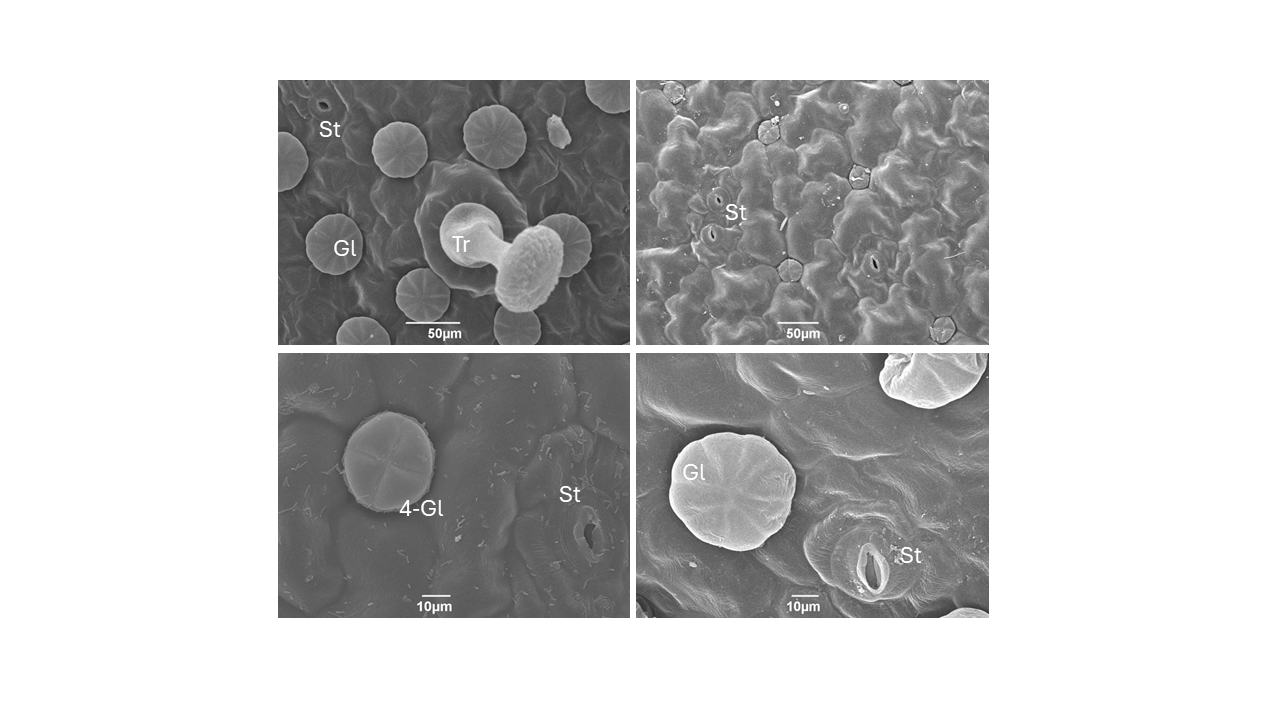


Plate 2 *Pinguicula leptoceras* Rchb.


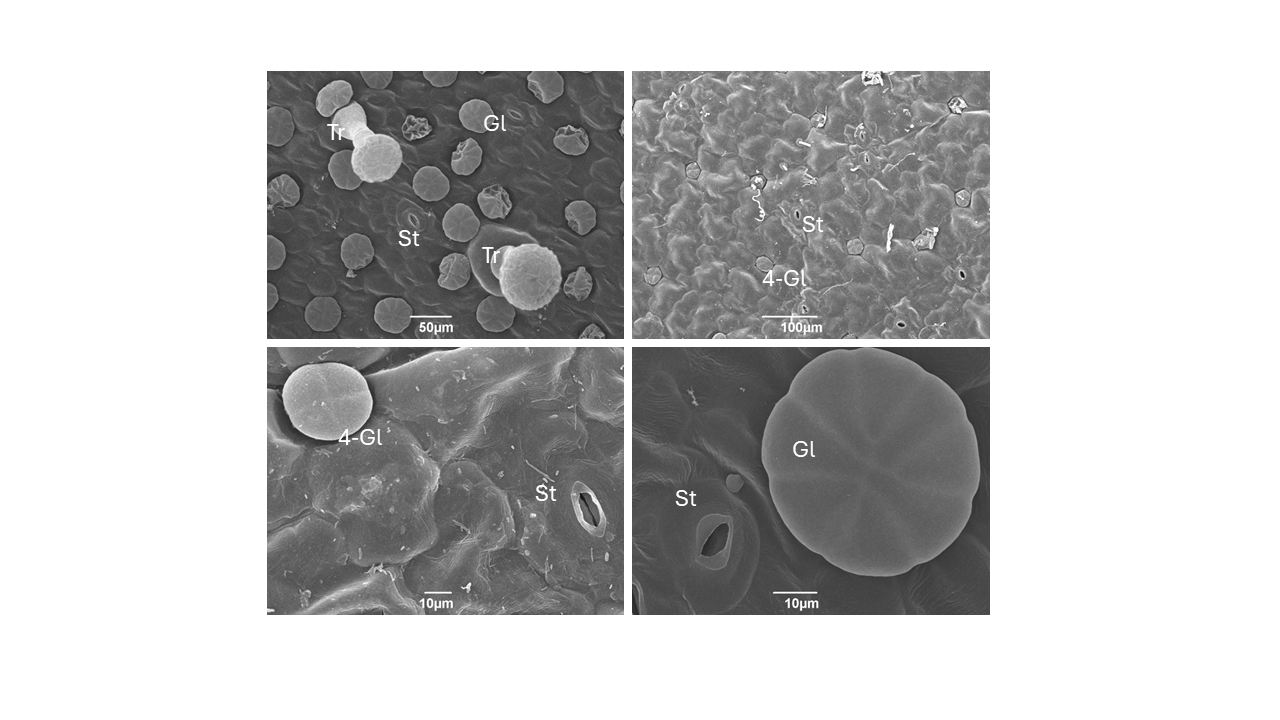


Plate 3 *Pinguicula poldinii* J. Steiger & Casper


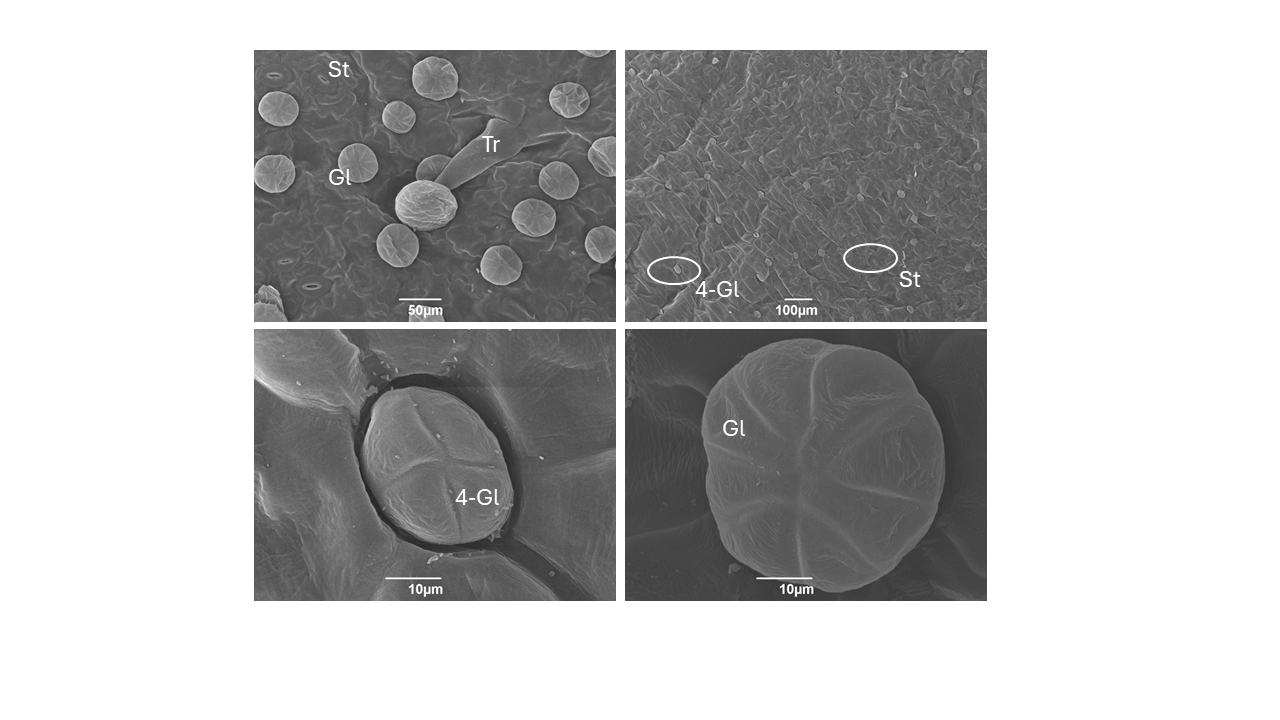


Plate 4 *Pinguicula vulgaris* L.


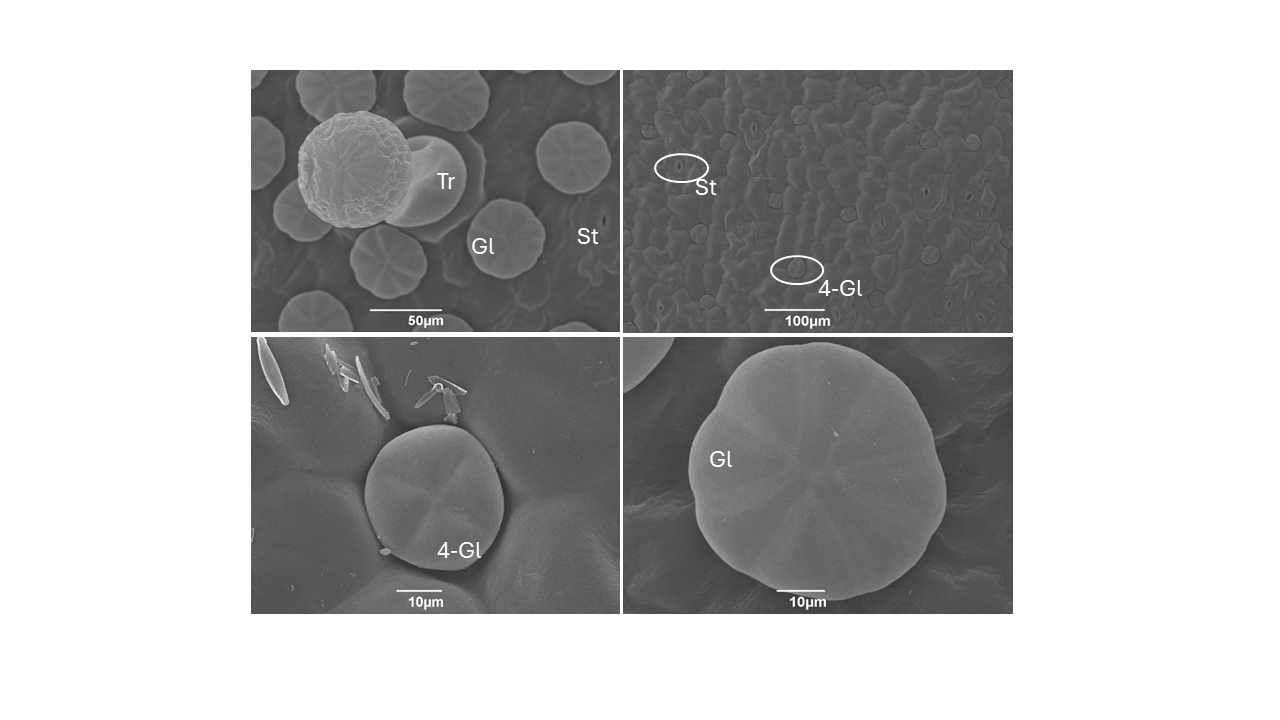

Supplement: Supplementary file 1 — Supplementary Material 1 [file 13104_2024_7021_MOESM1_ESM.docx]
